# Storing accepted scientific names alone can lead to misinterpretation of botanical data

**DOI:** 10.1038/s41598-026-47142-0

**Published:** 2026-04-22

**Authors:** Adam Richard-Bollans, Bob Allkin, Francesco Civita, Kristina Patmore, Rafaël Govaerts

**Affiliations:** Royal Botanic Gardens, Kew, Richmond, UK

**Keywords:** Taxonomic name resolution, Taxonomic databases, Taxonomy, World Checklist of Vascular Plants (WCVP), World Flora Online (WFO), Computational biology and bioinformatics, Ecology, Ecology, Evolution, Plant sciences

## Abstract

Botanists use accepted scientific names from authoritative databases to standardise taxonomic classifications. As taxonomic understanding evolves, these databases are updated, causing accepted names to change over time. A key decision when creating datasets about plants is whether to store the scientific names as originally recorded in a data source or to resolve them to currently accepted names. This decision partly depends on the *transitivity* of name resolution across taxonomic database versions: if name A resolves to B as the ‘accepted’ name in one version, and B to C in a later version, will A also resolve to C in that later version? Using the World Checklist of Vascular Plants and World Flora Online, we demonstrate that this transitivity fails for approximately 1% and 6% of names, respectively, and that the number of these discrepancies increases as new taxonomy versions are released. We recommend that botanical datasets store verbatim names as found and resolve them to accepted names as close to the point of end-use as possible, using the most recent taxonomic database.

## Introduction

Scientific names of plants change in response to new discoveries, new sources of data, revised classifications based on new analytical insights, and revised nomenclatural rules. There are, for example, about 10,000 changes to the scientific names of vascular plants published every year^1^. Taxonomic reference databases are consequently periodically updated to rearrange names to reflect their new taxonomic positions, mapping them to their corresponding accepted scientific names according to the latest taxonomic opinion. As a consequence, the ‘accepted’ scientific names assigned to taxa are liable to change over time to reflect the taxonomic position of each plant.

In parallel, many botanical datasets are continually being generated and updated to record, analyse and provide diverse insights into plants in a variety of domains. Such data often derive from previously published manuscripts, older datasets and specimens that may have been identified at different times. The creators and curators of these botanical datasets are faced with a choice when handling the names of taxa in their data – should they record and store the verbatim, original scientific names as found, i.e. including synonyms, misspellings etc.., or resolve those names to a current taxonomic database to retrieve the currently accepted scientific name?

The distinction between storing and disseminating verbatim or accepted names is often not considered or made explicit in data collection protocols. In the community curated dataset, WikiData^2^, synonymy is often resolved by users before inputting data. For example, the compound amsosinine is listed^1^ as occurring in *Amsonia elliptica*. This record is derived from a publication finding the compound occurring in *Amsonia sinensis*^3^, which is currently a synonym of *Amsonia elliptica* (Thunb.) Roem. & Schult.. In other cases, verbatim names are recorded as found and not resolved. For example, isovitexin is listed as occurring in *Gentiana orbicularis*^2^ from^4^, which is currently a synonym of *Gentiana brachyphylla* subsp. *favratii* (Rittener) Tutin. Some data sources are explicit in making name resolution part of the dataset creation process; for example, the Botanical Information and Ecology Network (BIEN) database^5^ resolves synonyms using the Taxonomic Name Resolution Service^6^ and releases data records under these accepted names. Other datasets, such as the Medicinal Plant Names Services (MPNS) dataset^7^ and TRY^8^, release data including accepted names of plants along with the original verbatim names as published.

When a scientific name is resolved to an accepted name, and the original name is discarded, information is lost regarding the description and taxonomic classification used in the original identification. In practice, this may have limited impact if the original and accepted names consistently resolve to the same name in future taxonomic databases. However, this depends on the *transitivity* of name resolution across versions of taxonomic databases i.e. given names *X*, *Y*, *Z* and taxonomic database versions *v*1, *v*2 such that:

$$X\xrightarrow[{v1}]{{{\mathrm{resolves}}\;{\mathrm{to}}}}Y\;{\mathrm{and}}\;Y\xrightarrow[{v2}]{{{\mathrm{resolves}}\;{\mathrm{to}}}}Z$$

is it the case that:

$$X\xrightarrow[{v2}]{{{\mathrm{resolves}}\;{\mathrm{to}}}}Z$$

If this property does not hold in general then creating datasets by storing only resolved, accepted names may create unexpected outcomes. In the above example, at the time of taxonomy version *v*2, if the name has been stored as *Y* then a researcher using the data would resolve this name to *Z*. If this transitivity property does not hold, then this would differ from the current understanding of how *X* should be resolved, i.e. to some other name, $Z'$. As far as we are aware, this transitivity property of taxonomic databases has not previously been analysed. The outcome of this has potentially wide-ranging consequences for botany and biological sciences more broadly, especially in an era where large scale data analyses are becoming increasingly prevalent in addressing many real-world challenges.

In this work we analyse this transitivity property for pairs of taxonomic database versions by comparing strategies that reflect different approaches to managing names in botanical datasets i.e. whether verbatim or accepted names are stored. We compare these approaches by assessing which names do not follow this transitivity property for a given pair of taxonomic databases and quantify the number of these discrepancies. To better understand where and when these discrepancies arise, we then investigate properties of the taxa and names related to these discrepancies. Firstly, we investigate the prevalence of homotypic and heterotypic synonyms involved in these discrepancies. We then quantify the number of synonymisations^3^ and resurrections^4^ between pairs of taxonomy versions and compare these to the number of resolution discrepancies. Next, we match the species involved in these intransitive resolutions to their geographic distributions, and highlight possible geographic biases. Finally, we discuss the implications of our findings and provide suggestions for how botanical dataset creators and curators should handle scientific names.

## Methods

To analyse this phenomenon for plant names in general we require large, authoritative taxonomies with accessible and consistent historical versions. For this we use both the World Checklist of Vascular Plants (WCVP)^9^, containing vascular plants, and World Flora Online (WFO)^10^, containing both bryophytes and vascular plants. This study is not intended as a comparison between the two, but to show that the phenomenon is not restricted to one specific taxonomy. Though both of these taxonomies are related, and so we might expect some duplication of results, there are important differences in their development and final output^11^.

Available versions of the WCVP were accessed using the *wcvpy* Python package^12^. This contains v10 (released October, 2022) along with biannual updates up to v14 (released May, 2025). Available versions of WFO were accessed manually through the Zenodo repository^13^. These comprise the earliest version released in July, 2018^14^ and eight periodic revisions up to December, 2024^15^.

### Quantifying discrepancies

Given an initial taxonomic database, $v_{1}$, and current taxonomic database, $v_{n}$, we consider the following strategies that reflect storage of scientific names in a dataset created at the time of $v_{1}$ and name resolution to $v_{n}$ by an end user of the data:

1. verbatim names are stored as found and resolved directly to $v_{n}$
2. names are resolved to $v_{1}$ and the accepted names are stored. These accepted names are then resolved to $v_{n}$
3. accepted names are periodically updated through each successive version of the taxonomy ($v_{1}$, $v_{2}$...$v_{n}$), rather than being resolved only once to $v_{n}$

For example, consider a name, *X*, of a plant to include in the dataset. Under strategy (1) this will be stored simply as *X*. Under strategy (2), this will be stored as the name that *X* resolves to in $v_{1}$, *Y*. At some time in the future, when $v_{n}$ is the current taxonomic database, the resulting name for strategy (1) is the resolution of *X* in $v_{n}$ and the resulting name for strategy (2) is the resolution of *Y* in $v_{n}$. Where these final names differ, we consider this as a discrepancy caused by storing accepted rather than verbatim names. We then assess if the observed discrepancies may be mitigated by strategy (3) of chaining the resolutions to accepted names through periodic updates of the stored accepted names i.e. $X\xrightarrow[{v1}]{{{\mathrm{resolves}}\;{\mathrm{to}}}}Y\xrightarrow[{v2}]{{{\mathrm{resolves}}\;{\mathrm{to}}}} \cdots \xrightarrow[{v_{{n - 1}} }]{{{\mathrm{resolves}}\;{\mathrm{to}}}}Y^{\prime } \xrightarrow[{v_{n} }]{{{\mathrm{resolves}}\;{\mathrm{to}}}}Y^{{\prime \prime }}$.

To quantify the likelihood of these discrepancies occurring across a pair of taxonomy versions, we begin by taking all taxon names from the earlier version (including authorship e.g. ‘*Artemisia abolinii* Lazkov’) that resolve to an accepted name. We then resolve these names to accepted names in the newer version following strategies (1), (2) and (3), and standardise this analysis across different taxonomy versions with different numbers of names by calculating the percentages of names in the original taxonomy for which a discrepancy (difference in final resolution between (1) and (2) or (1) and (3)) is found. We implement these analyses for the WCVP and WFO and report the number of names from the earliest taxonomy versions that resolved to different names, including how many of these resolve to different species and different genera. We include strategy (3) here in order to assess if this type of regular curation can help to avoid the discrepancies observed between (1) and (2).

To assess if the number of observed discrepancies between (1) and (2) tends to increase over time, for the oldest versions of the WCVP and WFO in our study we compare (1) and (2) using each more recent taxonomy version and assess the change in the percentage of discrepancies found using the Spearman correlation coefficient^16^. We use the Spearman coefficient as this is a non-parametric test that allows us to treat successive versions as ordinal data by release date. Due to the small number of data points, a permutation test with 9999 resamples is used to approximate the null distribution. Statistical significance is calculated using two-sided tests and all calculations are carried out using the scipy Python library^17^.

### Where do discrepancies arise from?

#### Homotypic & heterotypic synonyms

In general, we do not expect these types of discrepancies to arise from homotypic synonyms. Consider the motivating example above where $X\xrightarrow[{v1}]{{{\mathrm{resolves}}\;{\mathrm{to}}}}Y$. Suppose that in *v*1 this resolution occurs as *X* is a homotypic synonym of *Y*, i.e. that both names are associated with the same type specimen and that *Y* is considered the accepted name. For a discrepancy to arise where *X* and *Y* resolve to a different name in some later taxonomic version, *v*2 say, this would require two names with the same type specimen to be considered as referring to different taxon concepts. There are a few reasons this could occur, for example if it is decided that *X* and *Y* describe different taxonomic ranks for the same type specimen, or, of course, this could occur through simple error in the construction of the taxonomic database or record of the type specimens for both names. In contrast, if *X* is a heterotypic synonym of *Y*, i.e. both names are associated with different type specimens but are considered to resolve to the same taxon concept with accepted name *Y*, it may be considered at some future point in time that the two type specimens are distinct taxa, and create a resolution discrepancy.

To verify the assumption that this issue is mostly restricted to heterotypic synonyms, we count the heterotypic and homotypic synonyms involved in disagreements between strategy (1) and strategy (2) when considering v10 and v14 of the WCVP. We use the WCVP here as it includes consistent records of the homotypic and heterotypic status of synonyms.

#### Synonymisations & resurrections

To better understand the discrepancies we find, we compare these with the number of synonymisations and resurrections between pairs of taxonomy versions, using:

- The percentage of species names that are accepted in the old version that are not accepted in the new version (*synonymisations*)
- The percentage of species names that are not accepted in the old version that become accepted in the new version (*resurrections*)

We then analyse if the percentages of synonymisations and resurrections are correlated with species-level resolution discrepancies (for approaches (1) and (2)) for every pair of versions for each taxonomy, again using the Spearman correlation coefficient to statistically verify any relationship.

#### Geographic distribution

We also analyse if species from particular geographic regions tend to be involved more often in the discrepancies we find. This is an analysis we only conduct with the WCVP due to the availability of geographic distribution data for taxa in this taxonomy. First, we collect the number of native accepted species for each region according to WCVP v14, where regions are those according to the World Geographical Scheme for Recording Plant Distributions (Level 3)^18^. Then, using the analysis from Section Quantifying discrepancies, we collect those accepted species names that were involved in discrepancies at the level of species between strategies (1) and (2) when comparing WCVP v10 and v14, along with their native geographic distributions. We then compare the number of accepted species within a region against the number involved in a discrepancy.

To assess if any regions have a disproportionately high or low number of species involved in a discrepancy, we fit a LOWESS regression model^19^ which performs local linear fits and is robust to outliers. We highlight those regions where the residuals are greater than two standard deviations from the mean and also provide a visualisation of the global distributions of these residuals. The LOWESS model is implemented using the statsmodels Python library^20^.

## Results

### Resolution discrepancies

Results comparing disagreements between strategies (1) and (2) are given in Table 1. We can see that over approximately three years of WCVP versions around 1% of the original names resolve to different accepted names if following strategy (2) instead of strategy (1). Similarly, over approximately five years of WFO versions around 6% of names resolve to different accepted names.

**Table 1:** Disagreements between strategies (1) and (2) with oldest and newest taxonomy versions.

	WCVP 2022-10–2025-05	WFO 2018-07–2024-12
Original names	1359750	1051028
Total Disagreements	13303 (0.98%)	63676 (6.06%)
Species Disagreements	6826 (0.50%)	18911 (1.8%)
Genus Disagreements	718 (0.05%)	2741 (0.26%)

The breakdowns for how many of these discrepancies result in disagreements at the level of species and genera is also given. For example, if *Panicum nitidum* Lam. is resolved directly using WCVP v14, it resolves to *Dichanthelium nitidum* (Lam.) Mohlenbr. If this name is resolved using WCVP v10, it resolves to *Dichanthelium dichotomum* (L.) Gould., which then resolves to itself in v14 and so we end up with a difference in resolution at the level of species if the original name was stored as its accepted version in v10. For a genus-level example, *Vernonia setosquamosa* Hieron. resolves to itself in the 2024-12 version of WFO. If this name is resolved using the 2018-07 version of WFO, it resolves to *Lepidaploa remotiflora* (Rich.) H.Rob., which resolves to itself in the 2024-12 version of WFO; and so we end up with differences in genera i.e. *Vernonia* (approach 1) or *Lepidaploa* (approach 2). For both WCVP and WFO, the number of discrepancies that we see are not mitigated through strategy (3), i.e. periodic updates of data, see Table 2. These results provide a snapshot of discrepancies considering the earliest available and current versions of the WCVP and WFO taxonomies.

**Table 2:** Disagreements between strategy (1) and (3) with oldest and newest taxonomy versions.

	WCVP 2022-10–2025-05	WFO 2018-07–2024-12
Original names	1359750	1051028
Total Disagreements	13396 (0.99%)	61825 (5.88%)
Species Disagreements	6831 (0.50%)	20310 (1.93%)
Genus Disagreements	719 (0.05%)	3905 (0.37%)

**Fig. 1:**
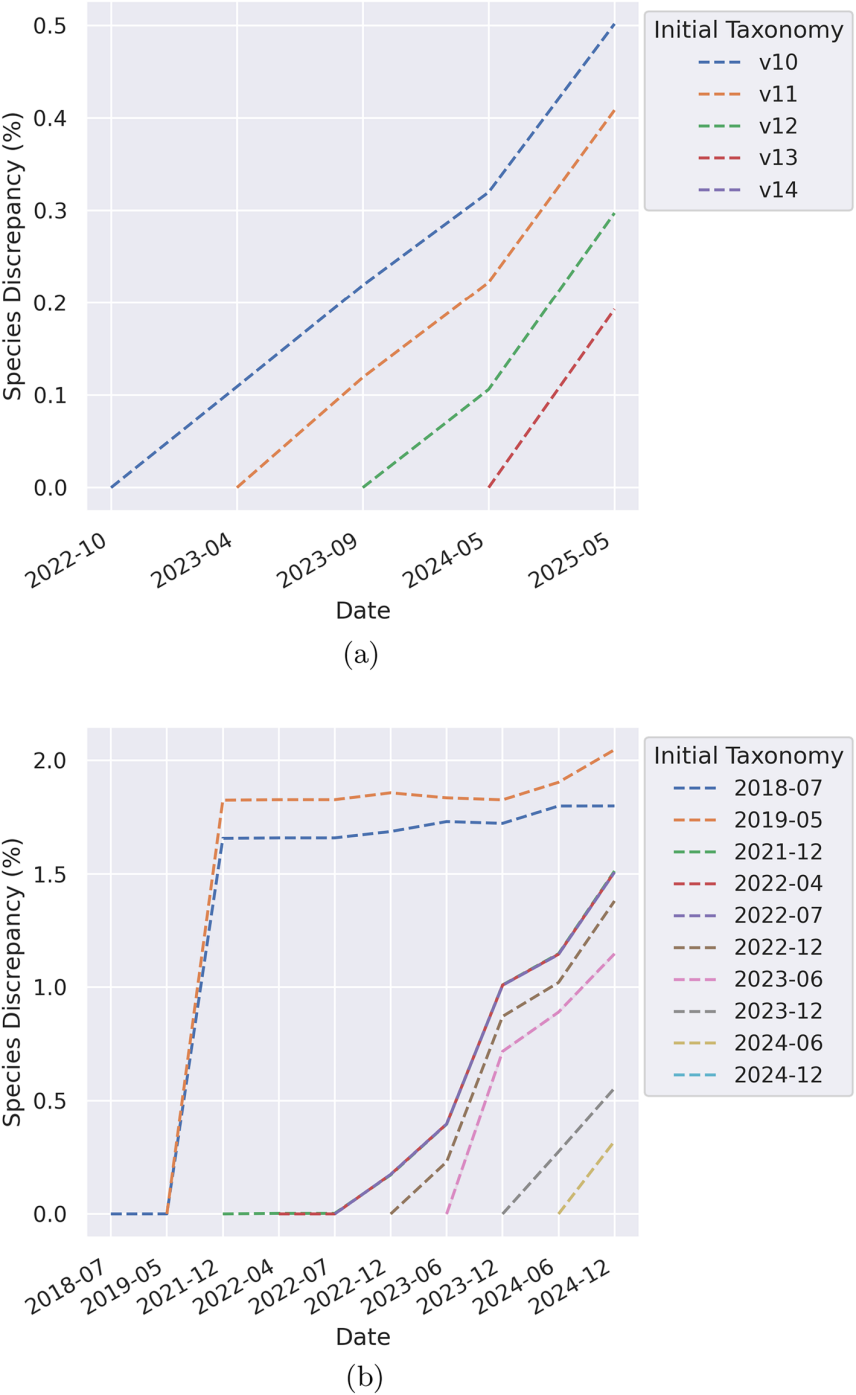
The increase of species-level resolution discrepancies over time between strategies (1) and (2), for different initial taxonomy versions.

Figure 1 shows how discrepancies may increase over time, specifically in the case of species disagreements. For a given initial taxonomy, the graph shows the percentage of names that resolve to a different species in future taxonomies if the names had been stored following strategies (1) and (2) (i.e. either as verbatim or as accepted names at the initial point in time). For example, using WFO, for a dataset constructed in 2018-07 around 1.8% of names are expected to differ at the level of species when resolved to the 2024-12 version if stored as accepted names instead of verbatim names. We can see that this final discrepancy falls as the initial versions become more recent, indicating that when a dataset is constructed using accepted names, the discrepancies increase over time.

For WFO in the 2018-07 case, the observed increase over time is found to be significant (p<0.001). For the WCVP v10 case, though the values are all monotonically increasing, this observation is not significant (p=0.08) due to the small sample size.

### Where do discrepancies arise from?

#### Synonym status

Of the 13303 disagreements reported in Table 1 between strategies 1 and 2 for v10 and v14 of the WCVP, 37 are from homotypic synonyms and 13266 are from heterotypic synonyms in v10. Of those discrepancies arising from homotypic synonyms, some result from differences in taxonomic ranks. For example, in v10 *Cassia acutifolia* Delile is a homotypic synoym of *Senna alexandrina* Mill.. In v14 *Cassia acutifolia* Delile resolves to *Senna alexandrina* var. *alexandrina*, while *Senna alexandrina* Mill. resolves to *Senna alexandrina* Mill.. Such a discrepancy may indicate a consistent use of the type specimen, e.g. that the original descriptions of *Cassia acutifolia* Delile and *Senna alexandrina* Mill. *do* refer to the same type specimen but that the type specimen is an example of the variety, described by *Cassia acutifolia* Delile, and that the description associated with *Senna alexandrina* Mill. describes the species concept. However, most of the discrepancies arising from homotypic synonyms do not fit this explanation. For example, *Dammara rumphii* C.Presl is a homotypic synonym of *Agathis dammara* (Lamb.) Poir. in v10. In v14 *Dammara rumphii* C.Presl resolves to *Agathis alba* (Rumph. ex Valmont) Oken, while *Agathis dammara* (Lamb.) Poir. resolves to *Agathis dammara* (Lamb.) Poir.. In this case, there is a single type specimen being used to describe two supposedly distinct species concepts. Nevertheless, the overwhelming majority of disagreements do arise from heterotypic synonyms, as expected.

#### Taxonomic changes

A breakdown of synonymisations and resurrections for species names for the earliest and current versions of the WCVP and WFO is given in Table 3. We can see that synonymisations are more common than resurrections and that these occur for a considerable number of names, especially for WFO.

**Table 3:** Breakdown of changes between oldest and newest taxonomy versions.

	WCVP 2022-10–2025-05	WFO 2018-07–2024-12
Accepted species names in old version	356292	371192
Synonymisations	7640 (2.14%)	45763 (12.33%)
Unaccepted species names in old version	667697	461146
Resurrections	3843 (0.58%)	11165 (2.42%)

**Fig. 2:**
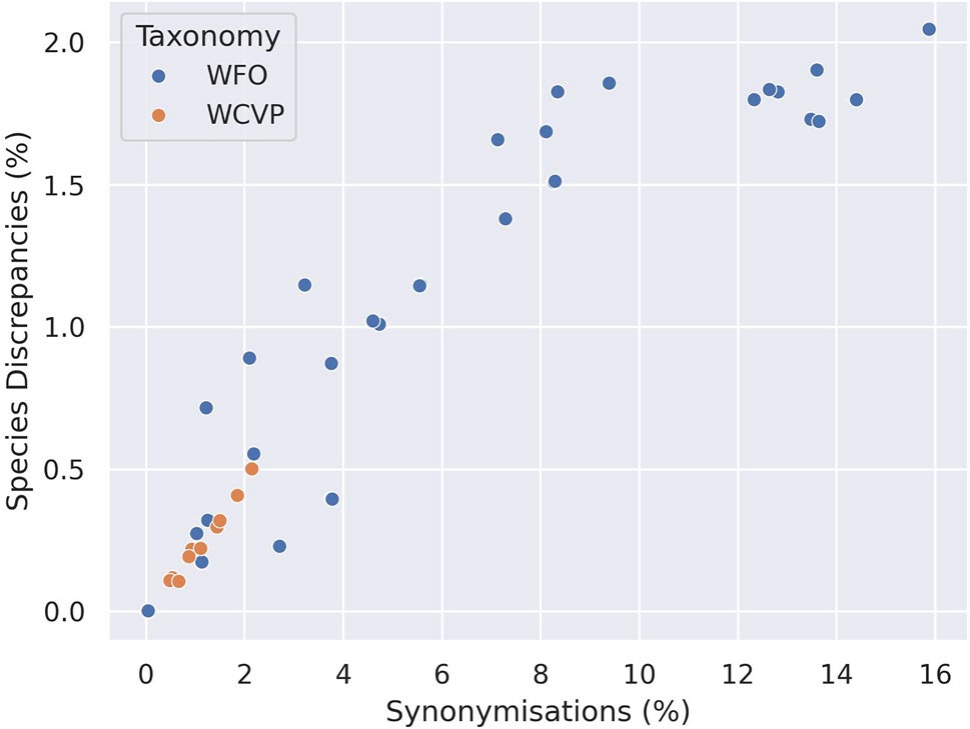
The relationships between disagreements in species-level resolution discrepancies for strategies (1) and (2), compared to the percentage of synonymisations.

**Fig. 3:**
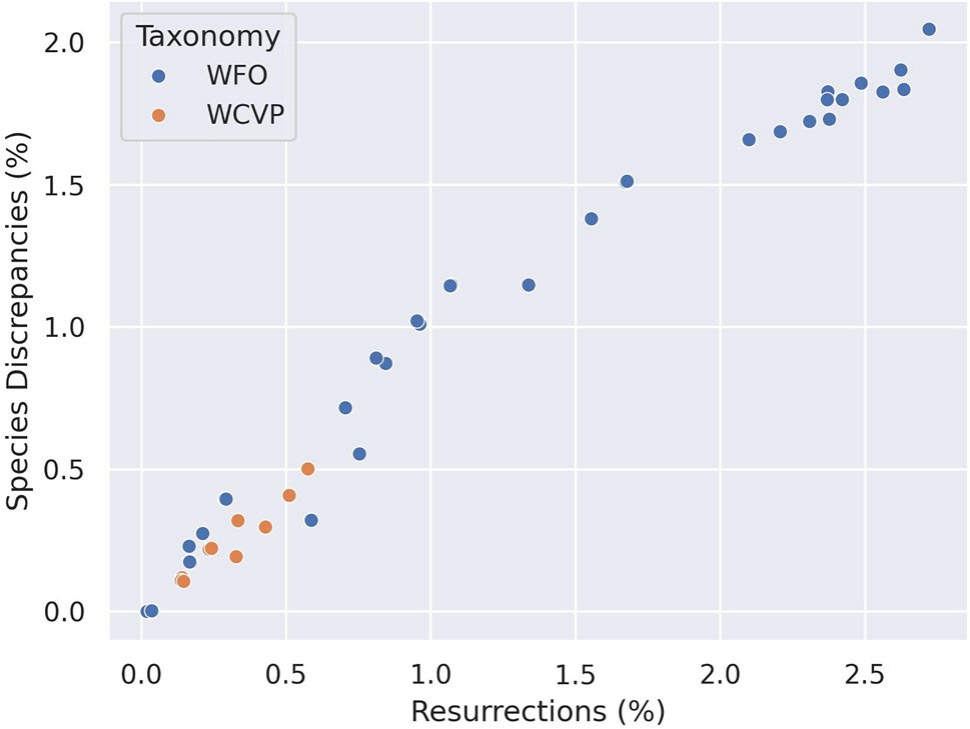
The relationships between disagreements in species-level resolution discrepancies for strategies (1) and (2), compared to the percentage of resurrections.

Figures 2 and 3 show the relationships between the percentages of synonymisations/resurrections and species-level discrepancies in name resolutions following approaches (1) and (2). In all cases, there is clearly a strong relationship (p<0.001). This is an unsurprising result, as one would expect more name changes overall to cause the kinds of discrepancies that we have been investigating, though there may still be confounding factors which require further exploration. For example, it may be the case that specific lineages are more challenging for taxonomists to agree on and this may cause both frequent name changes within taxonomies as well as discrepancies in name resolutions across taxonomy versions. Nevertheless, we believe this to be the first analysis quantifying synonymisation and resurrection rates for plant taxonomies, which may provide a starting point for future work on the topic.

#### Over and under-represented regions

**Fig. 4:**
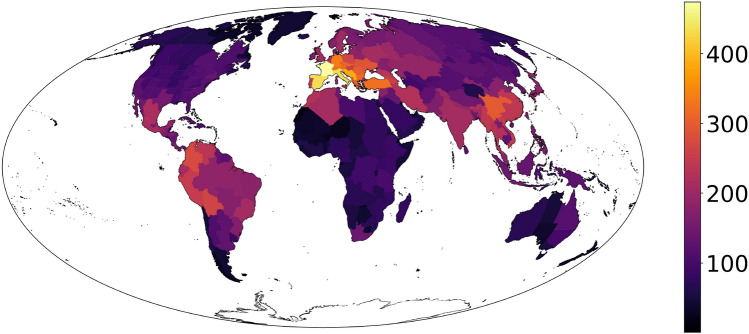
Global native distributions of accepted species that are incorrectly resolved to when following strategy (2) for WCVP versions v10 and v14.

**Fig. 5:**
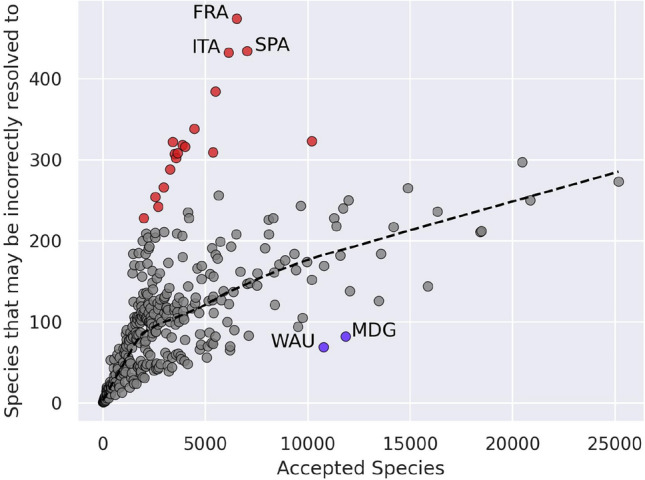
Number of species in a region vs number that are incorrectly resolved to following strategy (2). High and low-valued outliers highlighted in red and blue respectively. For readability only the top three high-valued outliers are annotated, the remaining outliers are: ALB, AUT, BGM, BUL, CZE, GER, GRC, HUN, POL, POR, ROM, SWI, TUR, UKR, YUG (for code definitions see^18^).

Figure 4 shows the global distribution of accepted species that are incorrectly resolved to when following strategy (2) (compared to (1)) for WCVP versions v10 and v14. Some of these regions have expectedly high counts due to the high number of species associated with these regions e.g. Colombia. However, there also seems to be unexpectedly high concentrations in Europe. To see this, we can plot these counts against the underlying number of native species in each region, as in Fig. 5. The LOWESS regression is also plotted here, which has an R$^{2}$ coefficient of determination of 0.43. The scatter plot identifies a number of over-represented regions (in red) with most arising from Europe and the most prominent being France, Spain and Italy. For these regions, this issue is particularly severe i.e. of the roughly 7000 species in France, around 500 could be incorrectly resolved to if a related data record had been stored using the accepted name according to v10. We can see that Madagascar and Western Australia are the only low-valued outliers, indicating relative taxonomic stability.

**Fig. 6:**
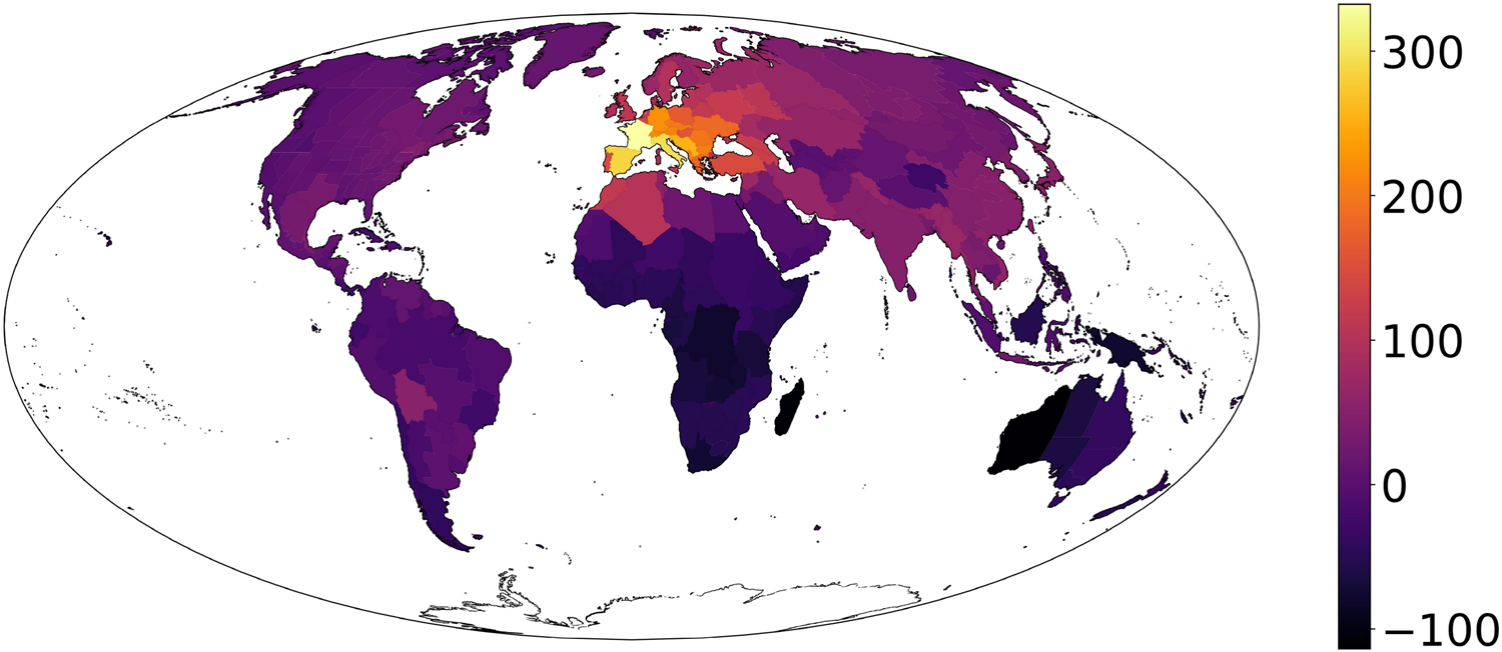
The global distribution of the residuals from the LOWESS regression.

To visualise the distribution of these over-represented regions at a global scale, a plot of the residuals is given in Fig. 6. This plot confirms the strong representation of regions in Europe. Greater ambiguity and instability of names in these regions may affect some global analyses of taxa in these regions, though this requires further investigation.

## Discussion

Large scale data analyses are becoming increasingly prevalent in addressing many challenges in botany and other biological sciences, with real-world impacts such as influencing conservation priorities^21^, estimating extinction risks^22^, or identifying potentially useful plant species^23^. There are a variety of botanical datasets used in these types of analyses and how names are managed may have significant impacts on outcomes. For example, the BIEN dataset resolves verbatim names to accepted names using the Taxonomic Name Resolution Service (TNRS) and releases data under these accepted names. At the time of writing, TNRS v5.3.1 may be used to resolve names to the WCVP or WFO using versions 12 and v.2023.06 respectively. Supposing these versions have been used to generate the present BIEN datasets and a researcher accesses this data and updates these names to WCVP v14 or WFO v.2024.12, we would expect this to create species-level resolution errors for around 0.3% and 1.14% of names, respectively. While we have only investigated this issue for plant names, we believe this is likely to apply more broadly e.g. to mycology and microbiology.

Another issue with storing accepted names is that the name resolution process must be carried out by the dataset creators. There are many ways to resolve scientific names^24^, many different taxonomic databases to resolve them to, and many pitfalls in the resolution process. When dataset creators tackle this problem, they enforce a particular resolution method and may obscure some of the ambiguities in the process. For example, during the resolution process one may encounter the name *Artemisia rupestris*, which, according to the WCVP, may correspond to any of eight homonyms (the same binomial published by different authors), each referring to separate distinct taxa. There are a variety of ways of handling this, e.g. by simply discarding such data, resolving the name to the genus *Artemisia* L., or by attempting to select the correct homonym based on other contextual information. Simply discarding the name may be appropriate in some contexts, such as in more generalised statistical analyses, but in others it may introduce undesired biases (e.g. species affected by these kinds of ambiguous homonyms are not evenly geographically distributed^5^). Lack of transparency regarding the methods used and any limitations will have inevitable but unseen implications for the end user.

In this paper we have investigated the specific issue of transitivity in the name resolution process, but the ambiguity of scientific names as identifiers in general has been well studied^25^, with suggested alternatives for managing taxon concepts in datasets^26,27^. However, it remains the case that names are commonly used as central identifiers linking data records to taxon concepts for botanical analyses.

There are many sources of uncertainty and inaccuracy in biological data that can have significant impacts on data analyses. Some sources of error or ambiguity are specific to certain data types, e.g. in occurrence records^28^, or may have broader implications, e.g. from misidentification of specimens^29^. The scientific names assigned to records in botanical datasets are fundamental identifiers for linking different properties of taxa within and across different datasets, and discrepancies in these key identifiers may have wide-ranging impacts. While it may not be feasible in every scenario to store the original verbatim names (i.e. names used to initially identify a specimen), where possible we strongly recommend basing datasets on these original names to minimise uncertainty and improve the quality of botanical analyses. In many cases, resolving names in datasets to accepted names is conducted in order to facilitate usage by end users. Where this is required, we recommend that (1) the original verbatim names are still provided in the dataset, (2) the accepted names are maintained and updated with each new taxonomic database version, and (3) the methods used to resolve names to accepted names are made clear and transparent to end users.

## Data Availability

All results and generated lists can be found in the Zenodo archive (https://doi.org/10.5281/zenodo.18082410) of the GitHub repository (https://github.com/alrichardbollans/SciNameTransitivity). Given two taxonomic database versions, v1 and v2, you can access a table of all names in v1 that resolve to different names in v2 using strategy (1) or (2) under the path [DB]_versions/outputs/v1_v2/all_results.csv, where [DB] is one of WCVP or WFO.

## Footnotes

1 https://www.wikidata.org/wiki/Q105289308 accessed 10/10/2025.

2 https://www.wikidata.org/wiki/Q3155636 accessed 10/10/2025.

3 Names that are accepted in an older taxonomy version that are not accepted in a new version.

4 Names that are not accepted in an older version that become accepted in a new version.

5 https://github.com/alrichardbollans/WCVPHomonyms

## Code availability

All collected data, analyses and results can be found in the Zenodo archive of the GitHub repository^30^.
